# Supplementary material for: Corneal confocal microscopy demonstrates minimal evidence of distal neuropathy in children with celiac disease
Source: PLoS One. 2020 Sep 21;15(9):e0238859. doi: 10.1371/journal.pone.0238859 (PMC7505458; doi:10.1371/journal.pone.0238859)
Supplement: Attachment — Submitted filename: Response to reviewers.pdf [file pone.0238859.s002.pdf]

August 13th, 2020
James Wolffsohn,
Academic Editor,
PLOS ONE

Re: PONE-D-20-16626 entitled "Corneal Confocal Microscopy Demonstrates Minimal Evidence of Distal Neuropathy in Children with Celiac Disease"

Dear Professor Wolffsohn,

Thank you for providing us with the opportunity to address the reviewers' concerns.
Please find attached a point-by-point response (in bold) to the concerns raised and a revised manuscript with the changes highlighted in red. We hope that you find our responses satisfactory and that the manuscript is now acceptable for publication.

Kind Regards,

Rayaz A Malik, MD, PhD
Professor of Medicine,
Weill Cornell Medicine-Qatar,
Qatar Foundation,
Education City,
PO Box: 24144-Doha, Qatar

Journal Requirements
Comment 1:<br>Please ensure that your manuscript meets PLOS ONE's style requirements, including those for file naming.<br><br>**Response 1:**<br>**The manuscript has been formatted according to PLOS ONE recommended style.**<br>**Changes are highlighted in Red.**
Comment 2:<br>Please provide additional details regarding participant consent. In the Methods section, please ensure that you have specified what type of consent you obtained (for instance, written or verbal) and whether the ethics committee approved this consent procedure. If verbal consent was obtained please state why it was not possible to obtain written consent and how verbal consent was recorded.<br><br>**Response 2:**<br>**Written assent and parental consent were obtained. This information is now stated on page 4, line 96 and IRB approval numbers are stated on page 5, line 98-99.**
Comment 3:<br>Thank you for stating the following in the Acknowledgments Section of your manuscript:<br>[This publication was made possible by a Sidra Internal Research Fund (SIRF) and a Biomedical Research Program [BMRP- 5726113101] grant from the Qatar National Research Fund (QNRF).]<br>We note that you have provided funding information that is not currently declared in your Funding Statement. However, funding information should not appear in the Acknowledgments section or other areas of your manuscript. We will only publish funding information present in the Funding Statement section of the online submission form.<br>Please remove any funding-related text from the manuscript and let us know how you would like to update your Funding Statement. Currently, your Funding Statement reads as follows:<br>[The funders had no role in study design, data collection and analysis, decision to publish, or preparation of the manuscript.]<br><br>**Response 3:**<br>**Thank you, this is now removed and funding statement.**<br>**We would like the funding statement to appear as follows:**<br>**This publication was made possible by a Sidra Internal Research Fund (SIRF) and a Biomedical Research Program [BMRP- 5726113101] grant from the Qatar National Research Fund (QNRF). The statements made herein are solely the responsibility of the authors.**
Comment 4:<br>Please include captions for your Supporting Information files at the end of your manuscript, and update any in-text citations to match accordingly. Please see our Supporting Information guidelines for more information: http://journals.plos.org/plosone/s/supporting-information.<br><br>**Response 4:**<br>**There is no supporting information associated with this manuscript other than the de-identified raw data.**
**Reviewer #1:**
Comment 1:<br>Abstract<br>The authors state that they are establishing the prevalence of small fibre damage and neuropathy in children with celiac disease. Given their sample size, are they confidently able to establish PREVALENCE?

Response 1:
Thank you for your kind feedback. We agree that a larger study is needed to assess the prevalence of neuropathy in children. We have therefore removed the word prevalence and simply indicated that we have assessed for the presence of neuropathy in this cohort of patients with CD.

Comment 2:
Introduction
The introduction is clear and well-written. I would again like to be convinced that what the authors are really doing is looking at prevalence. My understanding is that the authors are establishing CCM parameters in a group of children with celiac disease, compared to a group of controls. The presentation of the results does not support the aim of establishing prevalence. To do that, the authors would need to have an established cut-off for neuropathy and then establish neuropathy as the number of children with neuropathy as a proportion of the total number of children.

Response 2:
Thank you for raising this point. We agree that establishing prevalence requires a bigger sample size and reference /cut-off to compare to, thus we have rephrased the aim of this study. Please refer to page 2, line 30 and page 4 line 88.

Comment 3:
Methods
1. Please provide a sample size calculation. Lines 110-111 suggest that this is not possible, but I would argue that if you are detected neuropathy vs. no neuropathy, that there are many studies published that can provide means and standard deviations for this calculation.

Thank you, the sample size calculation has now been added on page 6, line 126-133.

2. Please explain how the six images were chosen e.g. were they overlapping by no more than 20% according to Vagenas et al?
Thank you, the criteria used for image selection has been added on page 5, line 114-116.

3. Why were corneal nerve parameters quantified manually using CCMetrics, rather than ACCmetrics?
Whilst the automated parameters measured using ACCmetrics correlate well with the manual CCMetrics, the actual value for CNFD, CNBD and CNFL is underestimated, especially for corneal nerve branch density. As this was the first study of CCM in CD, we have used manual analysis to ensure optimal accuracy, especially for the nerve fibre branch assessment.

4. How was corneal tortuosity established?
Corneal nerve fiber tortuosity is measured automatically with CCMetrics and is derived from the tracing of the main corneal nerve fibre. We have now clarified this on Page 5, lines118-119.

5. The authors provide no information on the CCM technique, or on the other clinical measurements taken
Additional details on the CCM and clinical tests have been added on page 4-5, line 96-99 and page 5, line 104-112.

Comment 4:
Results
1. Recommend the presentation of nerve data being to one decimal place – the additional decimal place does not add value. The same applies to p values, except where $p < 0.01$.
Thank you we have now modified accordingly.

2. The authors report correlations between corneal nerve features – I am not sure that this is required

given the original study aims – I cannot see how this adds value.
Thank you. Given the limited change in CCM parameters indicating minimal evidence of neurodegeneration in this cohort we wished to assess whether the reduction in nerve fibre length at the inferior whorl was related to the central CCM parameters, even though they were not lower. The correlations were also performed to gain insight into potential underlying associations between clinical and laboratory variables and CCM in CD.

3. Line 117 – please define OHD
OHD defined on page 7, line 144.

4. Line 136-7: suggest including the scatter plot of this significant correlation
Thank you, the scatter plot has been added.

Comment 5:
Discussion
I am concerned that the study was underpowered for the analysis undertaken
This is a pilot study. However, given that previous similar sized studies in children with diabetes and multiple studies in adults with other neurodegenerative conditions have shown significant corneal nerve loss we are confident that our findings indicate there is no significant neurodegeneration in children with CD as opposed to this study being underpowered. We have nevertheless acknowledged the possibility of the study being underpowered in the limitations. (Page 11 lines 229-230

Comment 6,7:
Figures
These are appropriate
Tables
These are appropriate

Response 6,7:
Thank you.

Reviewer #2

Comment 1:
1. The study presented CCM may serve as potential surrogate biomarker in detecting early sign of distal neuropathy in children with CD, and indeed, this has never been reported in the literature. Overall, the objectives, plans and methods carried out were coherent, and analysis employed was statistically sound. However, there are certain parts of the manuscript that require amendments and further clarification as listed below:
Thank you for the kind feedback.

2. Line 54: Insert 'and' between "osteoporosis, arthritis"
Inserted on page 3, line 57.

3. Line 81: Please define T1DM as it is the first time being introduced in the manuscript.
Defined on page 4, line 84-85.

Comment 2:
1. Line 91-92: Participant with "previous corneal trauma or systemic disease affecting the cornea" was excluded from the study. What about ocular disease/disorders/surgery; e.g. keratoconus, pellucid marginal degeneration, LASIK surgery etc? If excluded please include that in the exclusion criteria.
Thank you. We apply a standard list of exclusion criteria in all our studies and any systemic ocular disease ocular surgery and LASIK in the past 6 months are considered exclusion criteria for CCM. This information has been added on page 4, line 95.

2. CCM procedures: The authors did not explain about the process took place in acquiring CCM images. Instrument, type of scanning, method of scanning etc. If you have set a standard protocol, please explain the procedures prior to image selection and quantification section. If the protocol has been published, please cite the paper appropriately.
   Thank you, details of the CCM procedure have now been added on page 5, line 104-112.

3. Image selection and quantification: What was the process involved in selecting those CCM images? Were there any criteria? What were they?
   Thank you, the criteria for image selection are now added on page 5, line 114-122.

4. Image selection and quantification: I assume you selected 6 images to analyse CNFD, CNBD, CNFL & CNFT, and additional 6 images to analyse inferior whorl length? If Yes, please rephrase the sentence as it is a bit confusing.
   Thank you, the sentence is now rephrased on page 5, line 119-122.

5. Sample size: It is understandable that no prior data available for CCM in children with CD. However, could you please explain how you decide to examine 20 subjects in each group? What was the consideration?
   Thank you, the sample size calculation has been added on page 6. Line 127-134.

Comment 3:

1. Line 117: Define 25 OHD as it is the first time being introduced in the manuscript.
   Defined on page 7, line 144.

2. Line 121: Change (Fig 2A-D) to (Fig 2D), as this sentence only explains about CNFT, not other CCM parameters.
   Changed on page 7, line 149.

3. Table 1: Add "mean±SD" below Healthy (n=20) and CD (n=20) to make it easier for readers to understand rather than writing it in the description (line 126).
   Added, page 7, table 1.

Comment 4:

1. DISCUSSION: The first paragraph looks like a literature review rather than discussion. It is good to recap what has been previously reported, but please make sure it is not too lengthy. Perhaps, in the first paragraph, the authors can discuss about the results of the laboratory measures and relate with the current literature.
   Thank you, we have now changed the discussion as suggested to highlight the findings in relation to previous data as opposed to a literature review. Page 9 lines 176-181.
2. The study discussed/compared the CCM data with previous reports on diabetic patients. It is known both conditions have an impact on peripheral nervous system. Hence, it would be good to have an opening statement in the second paragraph of the discussion about similar effects of both conditions (i.e. neuropathy), and that observable in the corneal nerves using CCM.
   Thank you, added on page 10, line 197-198.

3. However, please avoid stating a direct comparison as these two conditions are not exactly the same, for example: Line 168-169 "This is in contrast with our previous studies showing a significant reduction in central corneal nerves in children with T1DM". I suggest use terms "similar trend" or "the results mimic".
   Thank you, this sentence has been rephrased on page 10, line 203-205.

4. Most CCM studies carried out on diabetic patients (cited in the manuscript) showed significant reduction in corneal nerve parameters. However, this was not the case for children with CD in

the present study (e.g. CNFD, CNBD & CNFL). Why corneal nerve degeneration in CD was less apparent? Discussion about this is crucial to the paper.
Thank you, further explanation has been added on page 10, line 203-210.

5. The study found CNFT was significantly lower in CD group compared to control. This is one of the hallmarks of the study but was not properly discussed (not even mentioned in the discussion). Please discuss what you think about it.
Thank you, we have now elaborated on CNFT on page 10, 207-210.

Reviewer #3

Comment 1:

This paper shows a very good review of the topic generally and it is very well documented! It also presents a very throughout design for measurement and wide knowledge on the examination process. Only additional point would be to consider to have further follow up visits or even looking into separate age groups, like young adults, adults, etc... to confirm if any evolution? - I do however feel, this point is a bit reflected at the end of the discussion when stating "Larger longitudinal studies are warranted..." Good job!

Response 1:

Thank you for the kind feedback.
